# Feasibility of Using Telehealth to Deliver the “Powerful Tools for Caregivers” Program

**DOI:** 10.5195/ijt.2017.6214

**Published:** 2017-06-29

**Authors:** KATRINA M. SERWE, GAYLE I. HERSCH, KAREN PANCHERI

**Affiliations:** 1 DEPARTMENT OF OCCUPATIONAL THERAPY, SCHOOL OF HEALTH PROFESSIONS, CONCORDIA UNIVERSITY, MEQUON, WISCONSIN, USA; 2 SCHOOL OF OCCUPATIONAL THERAPY, TEXAS WOMAN’S UNIVERSITY, HOUSTON, TEXAS, USA; 3 NELDA STARK COLLEGE OF NURSING, TEXAS WOMAN’S UNIVERSITY, HOUSTON, TEXAS, USA

**Keywords:** Caregivers, Feasibility studies, Health promotion, Telehealth, Telemedicine, Usability, Wellness

## Abstract

Caregivers report poorer health and wellness than the general population and identify numerous barriers to their attending programs to improve health and wellness. The purpose of this study was to explore the feasibility of employing a telehealth delivery method to enhance access to caregiver wellness programs. This article presents the quantitative results of a mixed methods feasibility study of translating the Powerful Tools for Caregivers (PTC) program to a telehealth delivery format. Four unpaid family caregivers of older adults participated in a telehealth delivered PTC program, a wellness program with established outcomes in the in-person environment. The program was delivered using synchronous videoconferencing methods. High class attendance and a high median total average Telehealth Usability Questionnaire score of 5.7 indicated the telehealth delivery method was feasible. This research suggests that telehealth is a feasible delivery format for a caregiver program traditionally delivered in an in-person format.

Caregivers are an important component of the health care system. Informal family caregivers of adults with chronic conditions provide unpaid care estimated at 470 billion dollars a year in the United States (Reinhard, Feinberg, Choula, & Houser, 2015). The demand for family caregivers is expected to increase as the population age 80 years and older rapidly grows (National Academies of Science, Engineering, and Medicine, 2016). Of great concern is that these caregivers are more likely to report health problems, stress, depression, and limited time to meet their own needs than the general population (Burton, Zdaniuk, Schulz, Jackson, & Hirsch, 2003; National Alliance for Caregiving, 2009; National Alliance for Caregiving & American Association for Retired Persons, 2015).

Caregiver wellness programs can address some of the issues caregivers face and prevent declines in their health. Powerful Tools for Caregivers (PTC) is one such program. PTC has demonstrated positive outcomes in the in-person format. Quasi-experimental trials of the PTC program demonstrate improved self-care behaviors (Boise, Congleton, & Shannon, 2005; Savundranayagam & Brintnall-Peterson, 2010; Won Won, Fitts, Favaro, Olsen, & Phelan, 2007), increased self-efficacy (Boise et al., 2005; Savundranayagam & Brintnall-Peterson, 2010), and increased use of community resources (Boise et al., 2005). Research also demonstrates reduced stress levels (Savundranayagam, Montgomery, Kosloski, & Little, 2011), reduced caregiver burden (Savundranayagam et al., 2011), and improved psychological well-being (Won Won et al., 2007). Participants who received the most benefits attended four or more of the six classes in the series. To their detriment, the participants who provided the most household help were less likely to complete the full PTC class series (Boise et al., 2005).

Caregivers with heaviest burdens were at the greatest risk for decreased health and wellness (Burton et al., 2003; Gallant & Connell, 1997. These caregivers experienced the most difficulty attending health and wellness programming. Full-time caregiving, lack of respite, and declining care receiver health are reasons caregivers have dropped out of in-person wellness programs (Kuhn, Fulton, & Edelman, 2003). Caregivers may face additional barriers to attending in-person programming, such as lack of time, lack of respite care, cost of respite care, difficulties with transportation, and costs associated with transportation. Some of these barriers can be addressed by delivering programs in the home via telehealth. Caregiver support groups and educational programs for caregivers have trialed telehealth delivery methods.

Past programs for caregivers designed specifically for telehealth delivery have employed a variety of delivery methods: telephone (Mahoney, Tarlow, Jones, Tennstedt, & Kasten, 2001; Marziali & Donohue, 2006; Tremont, Davis, Bishop, & Fortinsky, 2008), videophone (Bank, Arguelles, Rubert, Eisdorfer, & Czaja, 2006; Demiris, Oliver, Courtney, & Porock, 2005; Hanson & Clarke, 2000), internet (Brown, et al., 2016; Chiu et al., 2009; Demiris, Oliver, Wittenberg-Lyles, & Washington, 2011; Gallienne, Moore, & Brennan, 1993; Glueckauf, Ketterson, Loomis, & Dages, 2004; O’Connell et al., 2014; Savolainen, Hanson, Magnusson, & Gustavsson, 2008), and custom devices for communication and monitoring such as the Health Buddy 2.0 (Griffiths et al., 2010). This variety of interventions and outcome measures makes it difficult to compare these studies. However, overall, the research indicates that telehealth may be a successful delivery method for caregivers. Telehealth interventions have successfully reduced caregiver burden (Chiu et al., 2009; Glueckauf et al., 2004; Tremont et al., 2008), decreased symptoms of depression (Eisdorfer et al., 2003), improved self-efficacy (Glueckauf et al., 2004), improved decision making confidence (Gallienne et al., 1993), increased sense of security (Mahoney et al., 2001), improved quality of life (Demiris et al., 2005), decreased anxiety (Demiris et al., 2011), increased feelings of support (O’Connell et al., 2014; Savolainen et al., 2008) and improved knowledge and skill in caregiving tasks (Griffiths et al., 2010; Marziali & Donahue, 2006).

Telehealth delivery methods continue to improve. Past programs have primarily employed asynchronous methods, utilized synchronous methods without video components, or employed specialized equipment not available in a typical home. Programs that included synchronous audio and video delivery formats have not included synchronous audio and video connection between caregivers (Glueckauf et al., 2004), or have been delivered at a hospital-based site, not to a participant’s home (O’Connell et al., 2014). However, newer applications and greater availability of high speed internet options now make it possible to deliver services in a synchronous audio and video format similar to an in-person experience in the participant’s own home.

Transferring a successful in-person caregiver wellness program to the virtual environment via an in home synchronous videoconferencing telehealth method has not been tested. The goal of this study was to determine if a program traditionally delivered in the in-person environment can be accomplished via telehealth delivery methods. This article presents the quantitative results of a mixed methods feasibility study of translating the PTC program to a telehealth delivery format. Qualitative results are presented elsewhere (Serwe, 2016; Serwe, Hersch, Davel Pickens, & Pancheri, in press). The quantitative results presented here address the question: “Is telehealth in a synchronous videoconferencing format a feasible delivery method for a caregiver wellness program such as PTC?”

## METHOD

This feasibility study of a telehealth delivered PTC course engaged participants in a six-week program delivered using synchronous videoconferencing methods. Participants met in-person in their homes with the researcher the week before and the week after participation in the course. Participants completed initial assessments and received training in telehealth software use at the first meeting and completed post-assessments at the second meeting. This research was approved by the Texas Woman’s University Institutional Review Board and the Concordia University Institutional Review Board, and all participants provided informed consent prior to participation.

### PARTICIPANTS

Participants were recruited through purposeful advertisement of the program through a local area Aging and Disability Resource Center (ADRC). Inclusion criteria included: serving as an unpaid caregiver for an older adult who has a chronic condition; report of at least one barrier to attending an in-person program; ability to use a laptop computer and VSee® software; agreeable to meet with the researcher for an initial and post-intervention in-person meeting at the ADRC or the participant’s home; English speaking; and residence in southeastern Wisconsin. The primary investigator (PI) screened participants for eligibility both by phone and at the initial meeting.

Eight caregivers responded to the advertisement. Five caregivers signed up for the program, and one dropped out after the first PTC session, reporting that her caregiving situation was too different from the others. The final sample of four caregivers represented a convenience sample of eligible participants who responded to targeted advertisement of the study. All four participants were married, Caucasian, and lived in southeastern Wisconsin in a suburban or small town setting. Table 1 summarizes participant demographics, reported barriers to attending an in-person wellness program and caregiving situations.

### TELEHEALTH DELIVERY METHOD

The telehealth delivery method employed VSee^®^ software for synchronous videoconferencing. VSee^®^ is Health Insurance Portability and Accountability Act (HIPAA) compliant, adjusts to the available bandwidth by scaling down video quality to maintain call quality, and is free in the basic HIPAA Messenger version (VSee, 2017a; VSee 2017b). The software can display multiple people on a screen (i.e., sharing visual images and audio between all participants), and has a screen share feature. No more than six participants are recommended for optimal video streaming unless all participants have a high-speed connection (VSee, 2017b). The screen share feature allowed class leaders to display PTC PowerPoint slides and audio for all participants to view, without a need for participants to complete any additional computer actions on their device.

Participants 1 and 2 had laptop computers compatible with VSee^®^ software. Participants 3 and 4 were issued a loaned laptop, a 14-inch DELL^®^ Latitude E6410 computer with an Intel^®^ CORE i5 VPro processor and Windows 7^®^ operating system for the duration of the study. All participants had existing internet service in their homes.

The PI installed VSee^®^ software on the laptops of participants and guided participants through set up. Participants were issued a user guide for the software that was created specifically for this telehealth application. It contained a page to record login information, information on software use, information on troubleshooting potential technical difficulties, and contact information for the PI. Participants were trained in VSee^®^ software use at the in-person meeting with the user guide. Participants practiced basic functions required to participate in the telehealth program with minimal prompts from the PI including: opening the application, answering a call, and muting and unmuting audio and video.

### INTERVENTION

Participants met weekly for the PTC six-class series in a synchronous videoconferencing format. Each 90-minute PTC class teaches different “tools” for caregiving and participants have the option to read corresponding chapters in *The Caregiver Helpbook*, a reference text for the program (Boise et al., 2005). The PTC program delivery was as close to the traditional in-person format as possible. PTC is a scripted program; each class followed the PTC script. Visual materials were displayed on PowerPoint slides. Participants received a packet of handouts at the initial in-person meeting for reference throughout the class.

#### FIDELITY TO THE POWERFUL TOOLS FOR CAREGIVERS PROGRAM

Two aspects of the PTC program were changed to allow synchronous telehealth delivery. In the first PTC class, participants watch a twenty-minute video lecture. Streaming the video resulted in nonfunctional video quality in trial runs. Instead, participants viewed a static image of the lecturer and listened to the audio content of the video; this resulted in acceptable audio and video quality. Second, in-person PTC classes include paired partner discussion breakouts. Because this was not possible in the telehealth delivery format, all discussions involved the full group. While in-person PTC classes may have up to twelve participants, the telehealth format was limited to five participants because the selected software displays a recommended maximum of six video screens, with one of the video screens needed to display the class instructors.

### OUTCOME MEASURES

Participants completed a pre- and post-PTC survey of their demographics and caregiving situation. Class attendance was recorded, and caregivers completed the Telehealth Usability Questionnaire (TUQ) the week following the last PTC class. The TUQ measures usability of a telehealth system and has established reliability and validity (Parmanto, Lewis, Graham, & Bertolet, 2016). Participants also completed the Technology Profile Inventory, the Bakas Caregiving Outcomes Scale, and the Depression Anxiety Stress Scale, before and after PTC participation, and participated in a focus group the week following the last PTC class. The results not directly related to feasibility are presented elsewhere (Serwe, 2016; Serwe, Hersch, Davel Pickens, & Pancheri, in press).

### ANALYSIS

Microsoft Excel^®^ and SPSS^®^ version 22 software were used to summarize data with descriptive statistics. Results include: changes in the caregiving situation, class attendance, and telehealth system usability.

## RESULTS

Three out of the four caregivers reported a change in caregiving situation post-PTC participation (Table 1). Participants 1, 2, and 4 completed all six of the PTC classes. Participant 3 completed four of the six classes. She missed two classes due to commitments at her part-time job, but reported completing the corresponding chapter readings in *The Caregiver Helpbook* (PTC, 2013) to gain information presented in the missed classes.

### TELEHEALTH SYSTEM USABILITY

The TUQ provides information on usability of the telehealth system. TUQ total scores ranged from 5.0 to 6.4 on the seven-point scale, with a median total score of 5.7. A score of seven represents the most favorable score related to usability. Table 2 displays median scores and range for all participants on each item, subscale scores, and total score.

## DISCUSSION

This research explored the feasibility of delivering a traditionally in-person wellness program via telehealth synchronous delivery methods. Class attendance and TUQ scores informed the research question: “Is telehealth in a synchronous videoconferencing format a feasible delivery method for a caregiver wellness program such as PTC?” The delivery method proved feasible; the PTC class leaders delivered the six classes using VSee^®^ as scheduled with three of the four participants attending every class and the fourth missing two classes due to scheduled work.

### TELEHEALTH SYSTEM USABILITY

TUQ results indicate the telehealth PTC program was feasible. Participants viewed the method positively with a median total score higher than five out of seven. Previous telehealth feasibility studies employing the TUQ found ratings of five out of seven or higher in programs that proved feasible (Faett, Brienza, Geyer, & Hoffman, 2013; Parmanto, Pulantara, Schutte, Saptono, & McCue, 2013). Participants in this study also highly rated usefulness, ease of use, and satisfaction subscales, all with median scores higher than a six out of seven. Effectiveness and reliability subscales had the lowest ratings of the five subscales. The lowest scored items related to issues of audio feedback as indicated by item 12 “I can hear others clearly using the telehealth system” (University of Pittsburgh School of Health and Rehabilitation Sciences [UPSHRS], 2012) with a median score of 3.0 and consistently lower rating with a range of 3.0–4.0. However, participants indicated overall communication worked well as indicated by high ratings on item 11 “I can easily talk to other caregivers using the telehealth system with a median score of 5.5 and item 13 “I felt I was able to express myself effectively” (UPSHRS, 2012) with a median score of 6.5. The other low rated item, item 15 “I think the classes provided over telehealth are the same as in-person classes” (UPSHRS, 2012) had a wide range with one participant indicating complete disagreement with a rating of one and another participant rating with highest level of agreement with a seven. This variability in responses to this item indicates this may relate more to personal opinion than to the overall usability of the system. Item number 17 “The system gave error messages that clearly told me how to fix problems” (UPSHRS, 2012) also received a low rating with a median score of 3.0; however, only two participants rated this item. Two participants verbally told the PI at the final assessment that they did not rate this item because they did not encounter any errors. It is possible this item did not apply to the participants’ telehealth experience. Despite the lower ratings on the effectiveness and reliability subscales, all four participants reported they would use telehealth services again.

Personal opinion of telehealth and not encountering system errors may have decreased the TUQ total usability rating; however, the high median total score indicates participant agreement that the telehealth method employed was feasible for delivering the PTC program and overall the experience was positive.

### CAREGIVING SITUATION

Caregiving situation may affect participation rates. An e-mail based program found caregiver competence had an impact on participation rates, with lower levels of participation in caregivers who reported greater competence (Chiu & Eysenbach, 2010). A need for full time care and a decline in care receiver health are associated with attrition from caregiver wellness programs (Kuhn et al., 2003). However, in this study class attendance was high for all participants. The caregivers in this study were providing a relatively high level of care with three providing more than four hours of daily care and one providing one to two hours of daily care. One participant reported lack of respite as a barrier to participation in in-person programs and three reported barriers related to transportation. The barriers to in-person participation may have had an impact on the participants’ perceptions of the telehealth experience.

### LIMITATIONS AND CONCLUSION

The small sample size of four and lack of diversity in participants limits generalizability of results. Participants were Caucasian and from the same geographical area of southeastern Wisconsin. Participant level of education and some prior experience with computers for all participants may have influenced results.

The purpose of the study was to examine the feasibility of a telehealth delivered PTC program. Future research should examine the feasibility of the telehealth delivery format for caregivers from a variety of backgrounds and with caregivers who have no prior computer experience. Future research is also needed to determine best outcome measures to examine effectiveness of a telehealth delivered program.

## Figures and Tables

**Table 1:** Participant Demographics, Reported Barriers to Attending an In-Person Wellness Program, and Caregiving Situations

		Participant						
	
Characteristics		1		2		3	4	
Gender		F		F		F	M	
Age		61		69		67	83	
Employment		Works part-time		Homemaker		Works part-time	Retired	
Education		≥Master’s level		Bachelor’s degree		High school	Bachelor’s degree	
Class attendance barrier		Lack of respite care		Time related to transportation		Time related to transportation	Availability and cost of transportation	
Caregiving situation								
Relationship to care receiver		Daughter		Daughter		Spouse	Spouse	
Post-intervention living situation		Care receiver in care facility		Resides with care receiver		Resides with care receiver	Resides with care receiver	
	Pre	Post	Pre	Post	Pre	Post	Pre	Post
Daily hours of care	4+	2–3	4+	2–3	4+	4+	1–2	1–2
Type of care								
ADL	Yes	No	No	Yes	Yes	Yes	Yes	No
IADL	Yes	No	Yes	Yes	Yes	Yes	No	Yes

*Note*. ADL= activities of daily living; IADL = instrumental activities of daily living.

**Table 2:** Telehealth System Usability as Indicated by Telehealth Usability Questionnaire (TUQ) Results (n=4)

	Item	Median Score	Range (1–7)
1.	Telehealth improves my access to services, such as Powerful Tools for Caregivers.	6.0	(5.0–7.0)
2.	Telehealth saves me time traveling to get to services.	7.0	(5.0–7.0)
3.	Telehealth met my need to attend an educational program for caregivers.	6.5	(6.0–7.0)
	**Usefulness Scale Summary (Items 1–3)**	**6.5**	**(5.3–7.0)**
4.	It was simple to use this system.	6.0	(4.0–7.0)
5.	It was easy to learn this system.	6.5	(4.0–7.0)
6.	I believe I could become productive quickly using this system.	6.5	(6.0–7.0)
7.	The way I interact with this system is pleasant.	6.0	(4.0–7.0)
8.	I like using this system.	5.5	(4.0–7.0)
9.	The system is simple and easy to understand.	6.5	(4.0–7.0)
	**Ease of Use Scale Summary (Items 4–9)**	**6.3**	**(4.3–6.8)**
10.	This system is able to do everything I would want it to be able to do.	6.0	(4.0–7.0)
11.	I can easily talk to other caregivers using the telehealth system.	5.5	(4.0–7.0)
12.	I can hear others clearly using the telehealth system.	3.0	(3.0–4.0)
13.	I felt I was able to express myself effectively.	6.5	(5.0–7.0)
14.	Using the telehealth system, I can see others as well as if we met in person.	4.5	(3.0–7.0)
	**Effectiveness Scale Summary (Items 10–14)**	**4.9**	**(4.4–6.2)**
15.	I think the classes provided over telehealth are the same as in-person classes.	3.0	(1.0–7.0)
16.	Whenever I made a mistake using the system, I could recover easily and quickly.	6.0	(4.0–7.0)
17.	The system gave error messages that clearly told me how to fix problems.	3.0	(2.0–4.0)
	**Reliability Scale Summary (Items 15–17)**	**4.2**	**(3.0–6.0)**
18.	I feel comfortable communicating with others using the telehealth system.	6.0	(5.0–7.0)
19.	Telehealth is an acceptable way to receive services.	6.5	(5.0–7.0)
20.	I would use telehealth services again.	7.0	(7.0–7.0)
21.	Overall, I am satisfied with the telehealth system.	6.5	(6.0–7.0)
	**Satisfaction Scale Summary (Items 18–21)**	**6.5**	**(6.0–6.8)**
	**Total average**	**5.7**	**(5.0–6.4)**

*Note*. Two participants did not respond to item 17, as they did not find it relevant to them. All other items had a response from all four participants.
